# Effects of Frequency and Acceleration Amplitude on Osteoblast Mechanical Vibration Responses: A Finite Element Study

**DOI:** 10.1155/2016/2735091

**Published:** 2016-12-15

**Authors:** Liping Wang, Hung-Yao Hsu, Xu Li, Cory J. Xian

**Affiliations:** ^1^The Third Affiliated Hospital of Southern Medical University, Orthopaedic Hospital of Guangdong Province, Guangzhou 510630, China; ^2^Sansom Institute for Health Research, School of Pharmacy and Medical Sciences, University of South Australia, Adelaide, SA 5001, Australia; ^3^School of Engineering, University of South Australia, Adelaide, SA 5095, Australia

## Abstract

Bone cells are deformed according to mechanical stimulation they receive and their mechanical characteristics. However, how osteoblasts are affected by mechanical vibration frequency and acceleration amplitude remains unclear. By developing 3D osteoblast finite element (FE) models, this study investigated the effect of cell shapes on vibration characteristics and effect of acceleration (vibration intensity) on vibrational responses of cultured osteoblasts. Firstly, the developed FE models predicted natural frequencies of osteoblasts within 6.85–48.69 Hz. Then, three different levels of acceleration of base excitation were selected (0.5, 1, and 2 g) to simulate vibrational responses, and acceleration of base excitation was found to have no influence on natural frequencies of osteoblasts. However, vibration response values of displacement, stress, and strain increased with the increase of acceleration. Finally, stress and stress distributions of osteoblast models under 0.5 g acceleration in* Z*-direction were investigated further. It was revealed that resonance frequencies can be a monotonic function of cell height or bottom area when cell volumes and material properties were assumed as constants. These findings will be useful in understanding how forces are transferred and influence osteoblast mechanical responses during vibrations and in providing guidance for cell culture and external vibration loading in experimental and clinical osteogenesis studies.

## 1. Introduction 

It is widely accepted that bone is a dynamic tissue, because bone remodelling cells (including bone formation cells (osteoblasts) and bone degrading cells (osteoclasts)) can be activated under mechanical stimuli [1]. To analyse the exterior mechanical stimulation received by bone cells and their cellular responses, various mechanical stimuli have been used in* in vitro* studies since 1970 [2], for example, strain [3], fluid shear stress [4], and vibration [5]. An* in vivo* investigation of mice subjected to high-frequency mechanical signals suggested that some diseases or metabolic conditions can be inhibited or attenuated by vibrational stimuli, for example, adiposity [6]. Similarly, bone formation at the implantation sites and thus the osseointegration of bone-anchored implants can be enhanced by the vibrational stimuli [7, 8]. One* in vitro* sine-shaped vibration experiment with a displacement amplitude of 25 *μ*m and frequencies of 20–60 Hz applied to the cultured osteoblasts revealed that the vibration with an acceleration amplitude of 0.05 g and frequency of 20 Hz was optimal for cell proliferation and that the vibration with 0.13 g and 60 Hz was optimal for metabolic activity [9]. In a later study, a sinusoidal inertia force (at an acceleration amplitude of 0, 0.125 g, 0.25 g, or 0.5 g and frequency of 50 Hz) applied to cultured osteoblasts caused levels of gene expression of alkaline phosphatase (ALP) (a marker of osteogenic differentiation) to increase with the acceleration amplitude [10]. Furthermore, when MLO-Y4 osteocytes were exposed to low-magnitude, high-frequency vibration (0.3 g, 30, 60, and 90 Hz, 1 hour), their promoting effect on the osteoclast formation was inhibited [11]. These biomechanical experimental studies clearly illustrate that mechanical stimuli including vibration can affect bone cell formation and activity.

Human skeleton and bone cells are frequently subjected to vibration force experienced through activities or exercise, and the vibration is described often by frequency and by acceleration (acceleration < 1 g as low intensity and acceleration ≥ 1 g as high intensity) [12, 13]. While the acceleration values of human walking, running, and jumping hurdles are 1 g, 3-4 g, and 5 g, respectively [14], exercise can be considered as a repetitive vibration force with low frequency (~1-2 Hz) and high intensity [13]. Although the above and some other studies, using either experimental methods, computational methods, or finite element (FE) methods, have suggested that mechanical vibration can affect osteoblast proliferation, differentiation, and metabolic activity [9], as well as bone formation [7, 8], the biomechanical responses and the mechanisms of their responses of osteoblasts are unclear in responses to vibration stimuli of different acceleration and/or frequency.

In the present work, we aimed to investigate the biomechanical responses (displacement, von Mises stress, and strain) of osteoblasts of various shapes to mechanical vibration of different levels of acceleration. The main objectives of this study were fivefold: (1) to develop the idealized continuum FE models of osteoblasts of six different shapes; (2) to obtain the natural frequencies and mode shapes of all osteoblast FE models; (3) to determine the harmonic responses (like displacement and von Mises stress of nucleus centre) to base excitation vibration of osteoblast FE models; (4) to investigate the effect on osteoblast responses of base excitation under three levels of acceleration, that is, 0.5 g, 1 g, and 2 g, respectively; and (5) to investigate the effect factor on resonance frequency.

## 2. Materials and Methods

### 2.1. Geometry Information Used for Osteoblast FE Modelling

**Figure 1:**
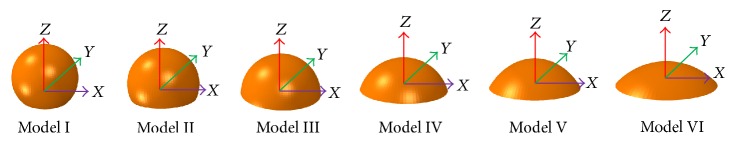
Geometry of idealized finite element models of osteoblasts of different shapes.

**Figure 2:**
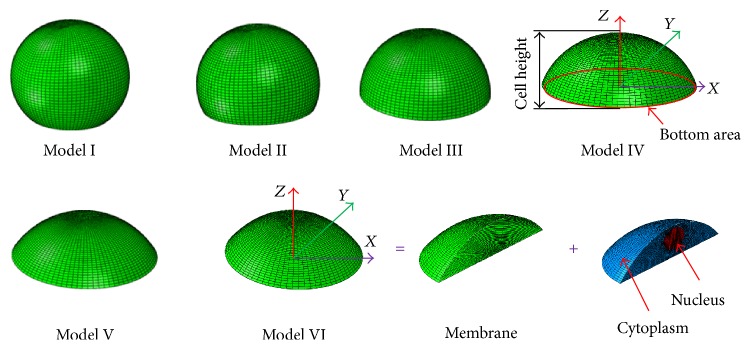
3D osteoblast finite element modelling.

The shapes of osteoblasts used for the FE modelling in the current study were from the results of experimental investigation [15] and computation modelling [16]. The osteoblast consists of three components, that is, the nucleus, cytoplasm, and cell membrane. In this geometry, the nucleus is embedded in the cytoplasm, the cytoplasm is between the nucleus and the cell membrane, and the cell membrane is the outermost surface of the whole cell (Figures 1 and 2). The fibrous materials (including microfilament and the microtubules) of the cell were neglected/ignored in all modelling in this present study. The geometry shapes and dimensions are shown in Figure 2 and Table 1.

The geometry of the osteoblasts is selected as part of a sphere. The nucleus is modelled as ellipsoid because the nucleus is normally modelled as sphere [17] or ellipsoid [16, 18, 19]. In this study, the volumes of the cell and nucleus are ~3000 *μ*m^3^ and 104.5 *μ*m^3^, respectively, which are based on the study of McGarry et al. [16]. The thickness of cell membrane is 6 nm [20, 21], and the endothelial cell membrane thickness also varies between 0.1 and 0.5 *μ*m [17]. The cell height is ~2–20 *μ*m [16, 21–23]. The bottom surface of the cell can be circle [16, 22–24] or ellipse [18, 19, 25] for the idealized models.

### 2.2. 3D Osteoblast FE Modelling

Here, the 3D osteoblast FE models were developed based on the corresponding geometry data. The FE models of different shapes are shown in Figure 1. Using software ABAQUS 6.14 (SIMULIA, Providence, RI, USA), eight-node hexahedral elements (C3D8) were used for the solid regions, that is, the nucleus and cytoplasm. Also, for the nucleus and cytoplasm, the hexahedral FE mesh was mapped according to the high quality geometries using eight-node C3D8 elements. At the same time, the cell membrane was meshed as shell element S4. The total element numbers of the nucleus, cytoplasm, and membrane were given in Table 1, respectively, for the six different models. To prevent any relative movement during subsequent vibration simulation analyses, in the FE modelling, the tie constraints were used to ensure attachment of the cytoplasm to the membrane and nucleus. In this study, we assumed there are no relative motions between cytoplasm and membrane and between cytoplasm and nucleus.

In this study, the materials were assumed isotropic/linear/elastic for the osteoblast FE models; and their properties and density for the osteoblast are given in Table 2. The density ratio 0.4 : 1 : 1.2 (600 kg/m^3^ : 1500 kg/m^3^ : 1800 kg/m^3^) of membrane, cytoplasm, and nucleus was assumed [26]. Young's modulus of membrane, cytoplasm, and nucleus was chosen at 1 kPa, 1.5 kPa, and 6 kPa, respectively, and Poisson's ratio was 0.3, 0.37, and 0.37 for membrane, cytoplasm, and nucleus, respectively.

For endothelial cells, Young's modulus of membrane, cytoplasm, and nucleus was set at 775 Pa, 775 Pa, and 5.1 kPa, respectively. Poisson's ratio of membrane, cytoplasm, and nucleus was set at 0.33 [27]. Normally, the elastic modulus of the cytoplasm is only one-quarter that of the nucleus [28]. It was reported that Young's modulus of the cytoplasm and nucleus was chosen at 100 Pa and 400 Pa, respectively, and Poisson's ratio was 0.37 for the cytoplasm and nucleus [16, 19, 29]. Previously, Young's modulus of 6.5 kPa and Poisson's ratio of 0.5 were assigned to the cytoplasm of an osteoblastic cell [22]. Elastic modulus of cytoplasm of osteocyte-like MLO-Y4 cell was set at 1.5 kPa [26]. Similarly, based on the measurements by atomic force microscopy, Young's modulus of 6 kPa was assigned to the osteoblast nucleus [30]. The elastic modulus of 1 kPa and Poisson's ratio of 0.3 were selected for the membrane of the adherent eukaryotic cell [16, 26].

Density is an important parameter in vibration simulation. The initial cellular density was assumed as 1000 kg/m^3^ [31], and 1250 kg/m^3^ was used as the density of cytoplasm, nucleus, and membrane in the endothelial cell [27]. Previously, the density of an osteoblast was assumed as 125 kg/m^3^ [19], and, for osteocyte-like MLO-Y4 cell, 1500 kg/m^3^, 1800 kg/m^3^, and 600 kg/m^3^ were set as the densities of cytoplasm, nucleus, and membrane, respectively [26].

### 2.3. Modal Analyses

#### 2.3.1. Natural Frequency Extraction

Natural frequency extraction is an eigenvalue analysis procedure, which determines the natural frequencies and shapes of mode for a structure. In this study, software ABAQUS was used to conduct the natural frequency extraction. The governing dynamic equation of the response in ABAQUS can be expressed as follows [32]:

(1) $$\begin{array}{c} M\ddot{u}+C\dot{u}+Ku=P, \end{array}$$

where *M*, *C*, and *K* (symmetric and positive definite) are the mass matrix, damping coefficient matrix, and spring stiffness matrix in the system, respectively. *P* is harmonic loading, and $\ddot{u}$, $\dot{u}$, and *u* are the acceleration vector, velocity vector, and displacement vector, respectively.

The free vibration structure without damping may be represented as

(2) $$\begin{array}{c} M\ddot{u}+Ku=0. \end{array}$$

The solution of *u* may be expressed as

(3) $$\begin{array}{c} u=\phi \sin\left(\;\omega t\;\right)\text{ or }\phi e^{i\omega t}. \end{array}$$

Then, (2) may be rewritten as

(4) $$\begin{array}{c} \left(\;-\omega^{2}M+K\;\right)\phi =0, \end{array}$$

where *ω* is the frequency and *ϕ* is the eigenvector (vibration mode).

Here, the natural frequencies of the six osteoblast models (Model I, Model II, Model III, Model IV, Model V, and Model VI) were obtained through the natural frequency extraction, and the corresponding mode shapes of the six osteoblast models were presented by the FE analysis.

#### 2.3.2. Harmonic Vibration

In this study, the free vibration of the system was considered with only one degree of freedom. In the simulation, the different levels of acceleration of base excitation were applied to analyse the effect of acceleration on the cell, that is, 0.5 g, 1 g, and 2 g (g = 9.8 m/s^2^), respectively. It is well known that acceleration (g forces, g = 9.8 m/s^2^) is the best term to describe vibration intensity [12]. In addition, the translational directions of bottom surface of FE models were constrained, which means that zero displacement was applied for the bottom surface of the cell model due to the fixed boundary condition.

#### 2.3.3. Effect Factor on Resonance Frequency

A previous human vibration test was conducted under the acceleration of 0.04–19.3 g, and it was found that the resonant frequency values of ankle, knee, hip, and spine are 10–40 Hz, 10–25 Hz, 10–20 Hz, and 10 Hz, respectively [33]. In this study, the effect of geometry on resonance frequency was investigated. The relationship between resonance frequency and the effect factor can be expressed through a fitted formula. The relationships of resonance frequency with cell height and with bottom area were analysed when the volume and density of the cell were assumed as constants. Cell height or cell bottom area can be considered as the effect factor, and the relationships of resonance frequency with cell height or with cell bottom area can be expressed as the formula based on the data of Figures 12(a) and 12(b).

## 3. Results 

### 3.1. Natural Frequency Extraction

**Figure 3:**
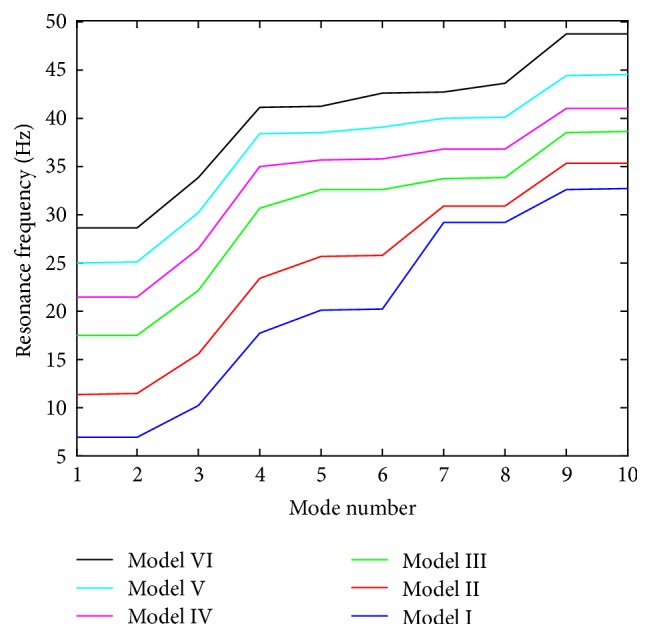
Resonance frequencies of the first ten modes for the 6 finite element models of osteoblasts.

**Figure 4:**
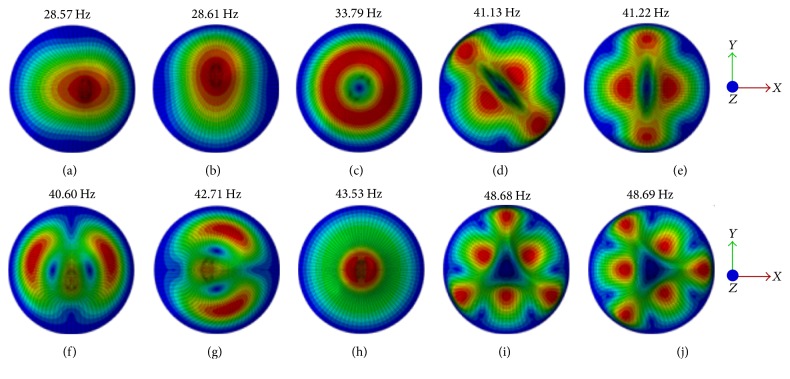
Mode shapes of the first ten vibration modes for Model VI (from top view). (a) 1st mode at 28.57 Hz; (b) 2nd mode at 28.61 Hz; (c) 3rd mode at 33.79 Hz; (d) 4th mode at 41.13 Hz; (e) 5th mode at 41.22 Hz; (f) 6th mode at 42.60 Hz; (g) 7th mode at 42.71 Hz; (h) 8th mode at 43.53 Hz; (i) 9th mode at 48.68 Hz; and (j) 10th mode at 48.69 Hz.

The natural frequencies and vibration mode shapes of the six osteoblast models were obtained after completing the FE modal analyses.  Figure 3 gives the resonant frequencies of the first ten modes for the six models. The first natural frequency values of osteoblasts for Model I–Model VI were 6.85, 11.35, 17.42, 21.40, 24.96, and 28.57 Hz, respectively. The corresponding mode shapes of Model VI are presented in Figure 4, and the natural frequencies for the first ten modes of Model VI were found at 28.57 Hz, 28.61 Hz, 33.79 Hz, 41.13 Hz, 41.22 Hz, 42.60 Hz, 42.71 Hz, 43.53 Hz, 48.68 Hz, and 48.69 Hz, respectively.

### 3.2. Responses to Harmonic Vibration

#### 3.2.1. The Displacement Response

**Figure 5:**
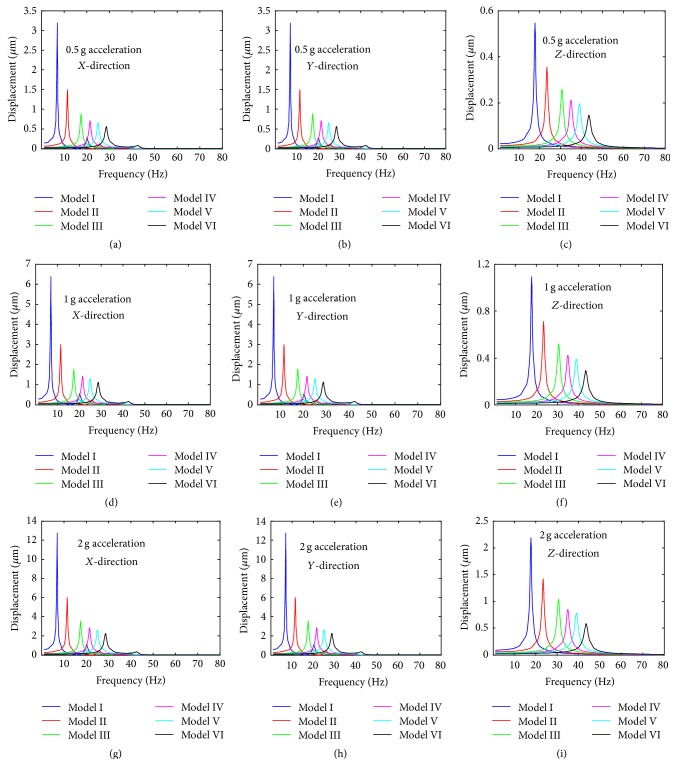
The displacement of the centre of the nucleus versus vibration frequency of osteoblasts. (a) 0.5 g acceleration in *X*-direction; (b) 0.5 g acceleration in *Y*-direction; (c) 0.5 g acceleration in *Z*-direction; (d) 1 g acceleration in *X*-direction; (e) 1 g acceleration in *Y*-direction; (f) 1 g acceleration in *Z*-direction; (g) 2 g acceleration in *X*-direction; (h) 2 g acceleration in *Y*-direction; and (i) 2 g acceleration in *Z*-direction.

Based on the natural frequencies and the assumed uniform acceleration of base excitation within the frequency range between 1 and 50 Hz, the harmonic responses of the six FE models were computed. To investigate the responses of the different directions (*X*-, *Y*-, and *Z*-directions) to the harmonic vibration, the displacement values at the centre of the nucleus (Figure 1) were plotted in Figure 5 for the different levels of acceleration (0.5 g, 1 g, and 2 g). The frequency values of the peak displacement in *X*-direction for Model I–Model VI are 6.85 Hz, 11.35 Hz, 17.42 Hz, 21.40 Hz, 24.96 Hz, and 28.57 Hz, respectively. The frequency values of the peak displacement in *Y*-direction for Model I-Model VI are 6.88 Hz, 11.38 Hz, 17.46 Hz, 21.44 Hz, 25.00 Hz, and 28.61 Hz, respectively. The frequency values of the peak displacement in *Z*-direction for Model I–Model VI are 17.69 Hz, 23.38 Hz, 30.59 Hz, 34.97 Hz, 38.99 Hz, and 43.51 Hz, respectively.

**Figure 6:**
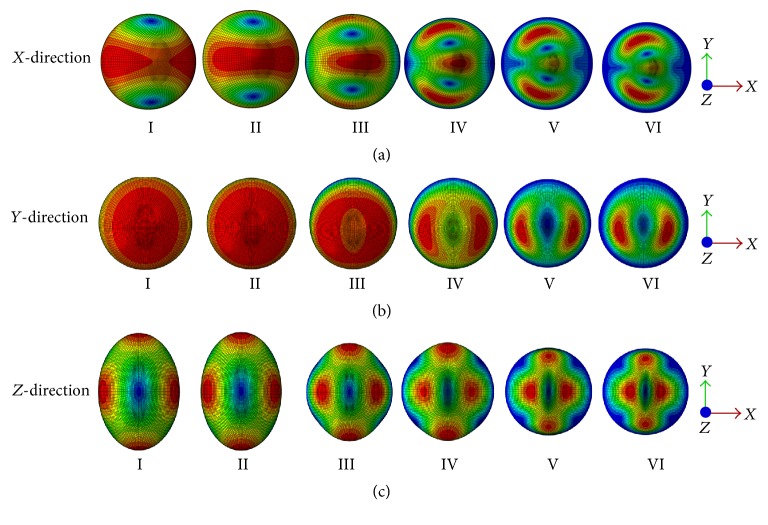
Mode shapes at the peak frequency under 0.5 g acceleration (from top view). (a) In* X*-direction; (b) in* Y*-direction; and (c) in* Z*-direction.

**Figure 7:**
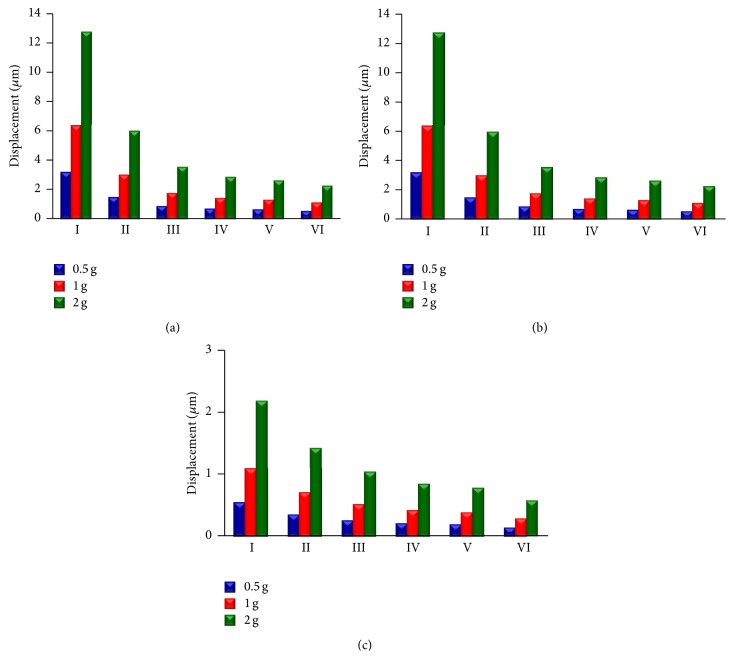
Displacement distribution at the peak frequency under different levels of base excitation acceleration. (a) In *X*-direction; (b) in *Y*-direction; and (c) in *Z*-direction.

The mode shapes of the FE models at the peak frequency under 0.5 g acceleration in *X*-direction, *Y*-direction, and *Z*-direction are presented in Figures 6(a), 6(b), and 6(c), respectively. In addition, the displacement values of the models under the different levels of base excitation acceleration in *X*-direction, *Y*-direction, and *Z*-direction are given in Figure 7. The order of displacement of the centre of the nucleus for the different acceleration in *X*-direction, *Y*-direction, and *Z*-direction is 2 g > 1 g > 0.5 g. The order of displacement of the centre of the nucleus for the different models in *X*-direction, *Y*-direction, and *Z*-direction is Model I > Model II > Model III > Model IV > Model V > Model VI. The lowest displacement values of the centre of the nucleus for Model VI under 0.5 g acceleration in *X*-direction, *Y*-direction, and *Z*-direction are 0.565 *μ*m, 0.565 *μ*m, and 0.146 *μ*m, respectively. The largest displacement values of the centre of the nucleus under 2 g acceleration in *X*-, *Y*-, and *Z*-direction are 12.749 *μ*m, 12.756 *μ*m, and 2.188 *μ*m, respectively.

#### 3.2.2. The von Mises Stress and the Strain

**Figure 8:**
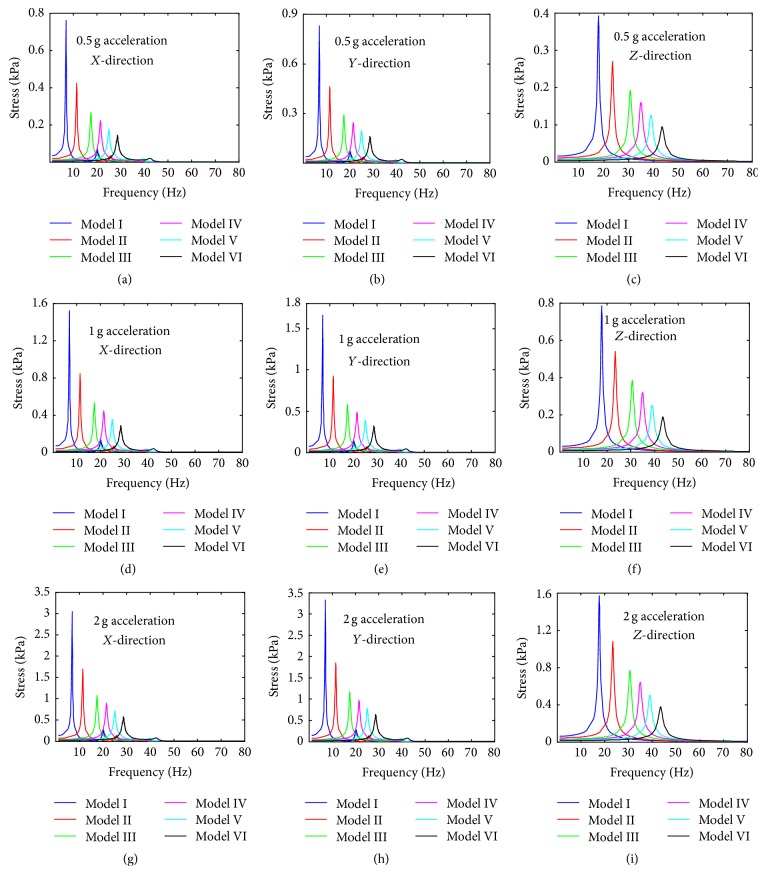
The von Mises stress value of the centre of nucleus versus frequency under different levels of acceleration. (a) 0.5 g acceleration in *X*-direction; (b) 0.5 g acceleration in *Y*-direction; (c) 0.5 g acceleration in *Z*-direction; (d) 1 g acceleration in *X*-direction; (e) 1 g acceleration in *Y*-direction; (f) 1 g acceleration in *Z*-direction; (g) 2 g acceleration in *X*-direction; (h) 2 g acceleration in *Y*-direction; and (i) 2 g acceleration in *Z*-direction.

In addition, the von Mises stress values of the centre of the nucleus under the different levels of base excitation in *X*-, *Y*-, and *Z*-directions are given in Figure 8. The lowest von Mises stress values of Model I under 0.5 g in *X*-, *Y*-, and *Z*-directions are 1 for all of them. The order of von Mises stress values of the centre of the nucleus for the different base excitation in *X*-, *Y*-, and *Z*-directions is 2 g > 1 g > 0.5 g. The order of von Mises stress values of the centre of the nucleus for the different models in *X*-, *Y*-, and *Z*-directions is Model I > Model II > Model III > Model IV > Model V > Model VI. The lowest von Mises stress values of the centre of the nucleus for Model VI under 0.5 g in *X*-, *Y*-, and *Z*-directions are 0.145 kPa, 0.16 kPa, and 0.094 kPa, respectively. The largest von Mises stress values of the centre of the nucleus for Model I under 2 g in *X*-, *Y*-, and *Z*-directions are 3.05 kPa, 3.33 kPa, and 1.57 kPa, respectively.

**Figure 9:**
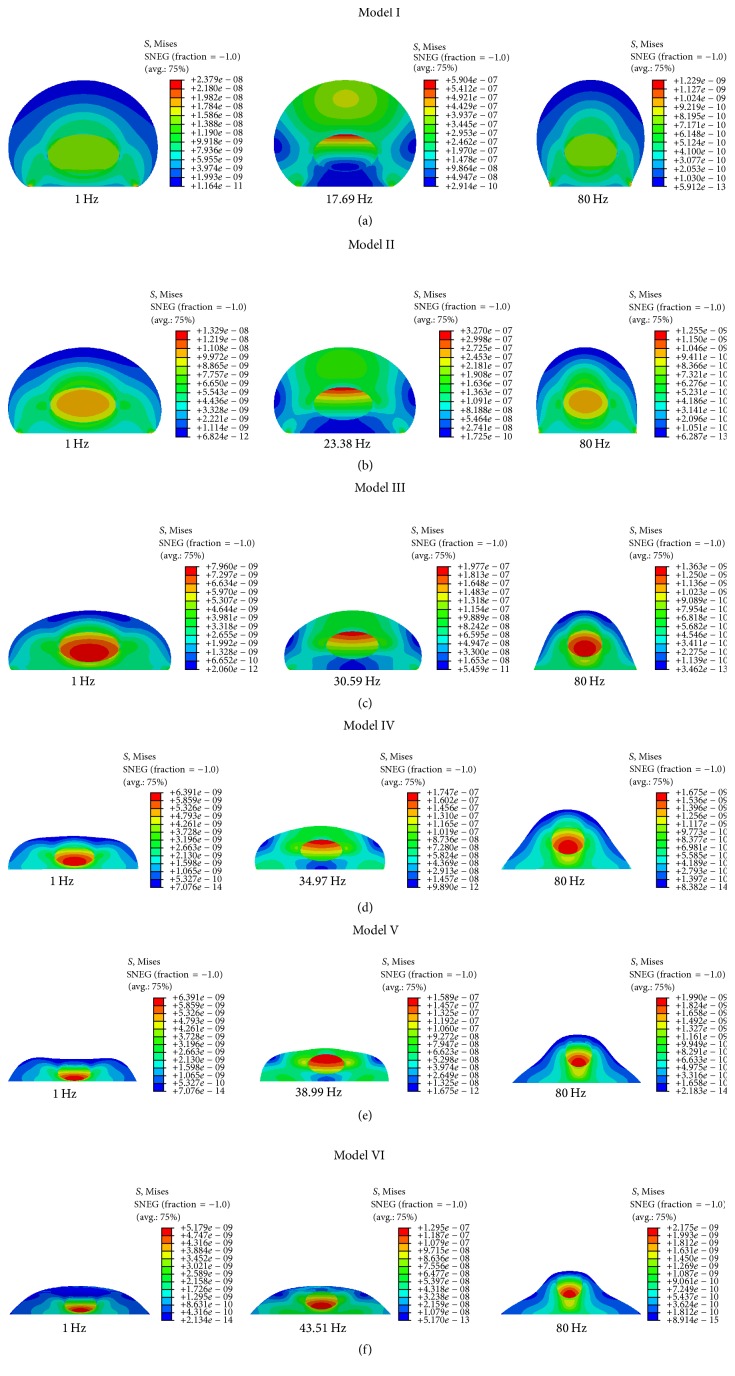
von Mises stress contours of the different cell models in *Z*-direction under 0.5 g base excitation. (a) Model I; (b) Model II; (c) Model III; (d) Model IV; (e) Model V; and (f) Model VI.

Moreover, the von Mises stress contours of the FE models under 0.5 g base excitation in *Z*-direction at the frequency of 1 Hz, peak value, and 80 Hz are shown in Figure 9. The peak frequency values in *Z*-direction for Model I–Model VI are 17.69 Hz, 23.38 Hz, 30.59 Hz, 34.97 Hz, 38.99 Hz, and 43.51 Hz, respectively. The maximum von Mises stress values of the cell at the peak frequency for Model I–Model VI under base excitation in *Z*-direction are 590.4 Pa, 327 Pa, 197.7 Pa, 174.7 Pa, 158.9 Pa, and 129.5 Pa, respectively. At 80 Hz, the maximum von Mises stress values are 1.229 Pa, 1.255 Pa, 1.363 Pa, 1.675 Pa, 1.99 Pa, and 2.175 Pa, respectively.

**Figure 10:**
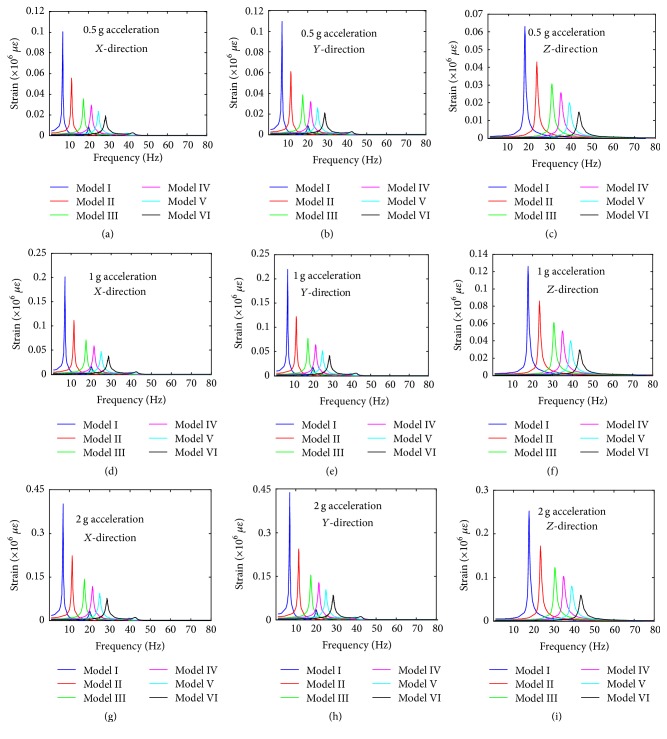
The strain value of the centre of nucleus versus frequency of the six different cell models at different levels of acceleration. (a) 0.5 g acceleration in *X*-direction; (b) 0.5 g acceleration in *Y*-direction; (c) 0.5 g acceleration in *Z*-direction; (d) 1 g acceleration in *X*-direction; (e) 1 g acceleration in *Y*-direction; (f) 1 g acceleration in *Z*-direction; (g) 2 g acceleration in *X*-direction; (h) 2 g acceleration in *Y*-direction; and (i) 2 g acceleration in *Z*-direction.

Similarly, the strain values of the centre of the nucleus under the different base excitation in *X*-, *Y*-, and *Z*-directions are given in Figure 10. The lowest strain values for Model I under 0.5 g in *X*-direction, *Y*-direction, and *Z*-direction are 1 for all of them. The order of strain values of the centre of the nucleus for the different base excitation in *X*-direction, *Y*-direction, and *Z*-direction is 2 g > 1 g > 0.5 g. The order of strain values of the centre of the nucleus for the different models in *X*-, *Y*-, and *Z*-directions is Model I > Model II > Model III > Model IV > Model V > Model VI. The lowest strain values of the centre of the nucleus for Model VI under 0.5 g in *X*-, *Y*-, and *Z*-directions are 19095.4, 21096.5, and 14827.6 microstrains, respectively. The largest strain values of the centre of the nucleus for Model I under 2 g in *X*-, *Y*-, and *Z*-directions are 401550, 438575, and 252340 microstrains, respectively.

**Figure 11:**
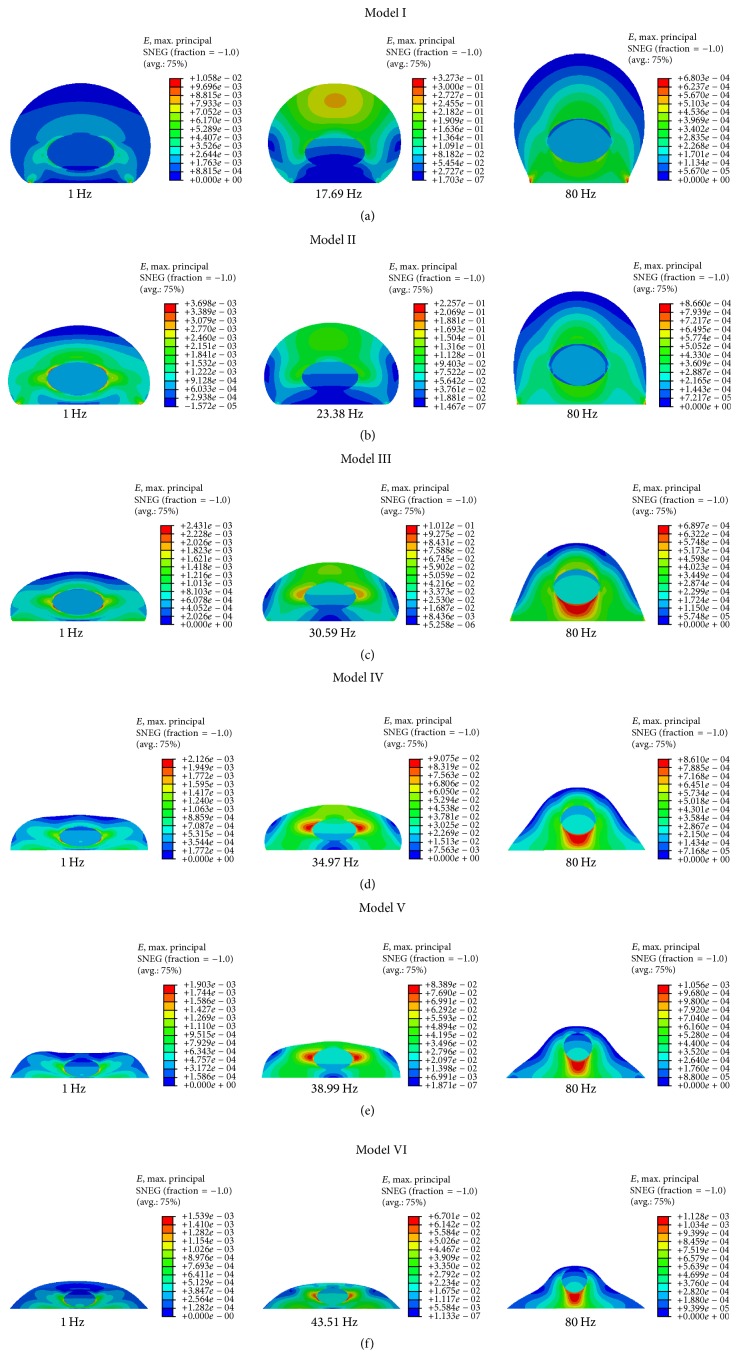
Strain contours of the cell in *Z*-direction under 0.5 g base excitation. (a) Model I; (b) Model II; (c) Model III; (d) Model IV; (e) Model V; and (f) Model VI.

Furthermore, the strain contours of the 6 FE models under 0.5 g base excitation in *Z*-direction at the frequency of 1 Hz, peak value, and 80 Hz are given in Figure 11. The maximum strain values of the cell for Model I–Model VI in *Z*-direction at the peak frequency are 327300, 225700, 101200, 90750, 83890, and 67010 microstrains, respectively. There is a fluctuation of the maximum strain value of the cell at the peak frequency. At 80 Hz, the maximum strain values are 680.3, 866, 689.7, 860.1, 1056, and 1128 microstrains, respectively.

### 3.3. Effects of Cell Height and Bottom Area on Resonance Frequency

**Figure 12:**
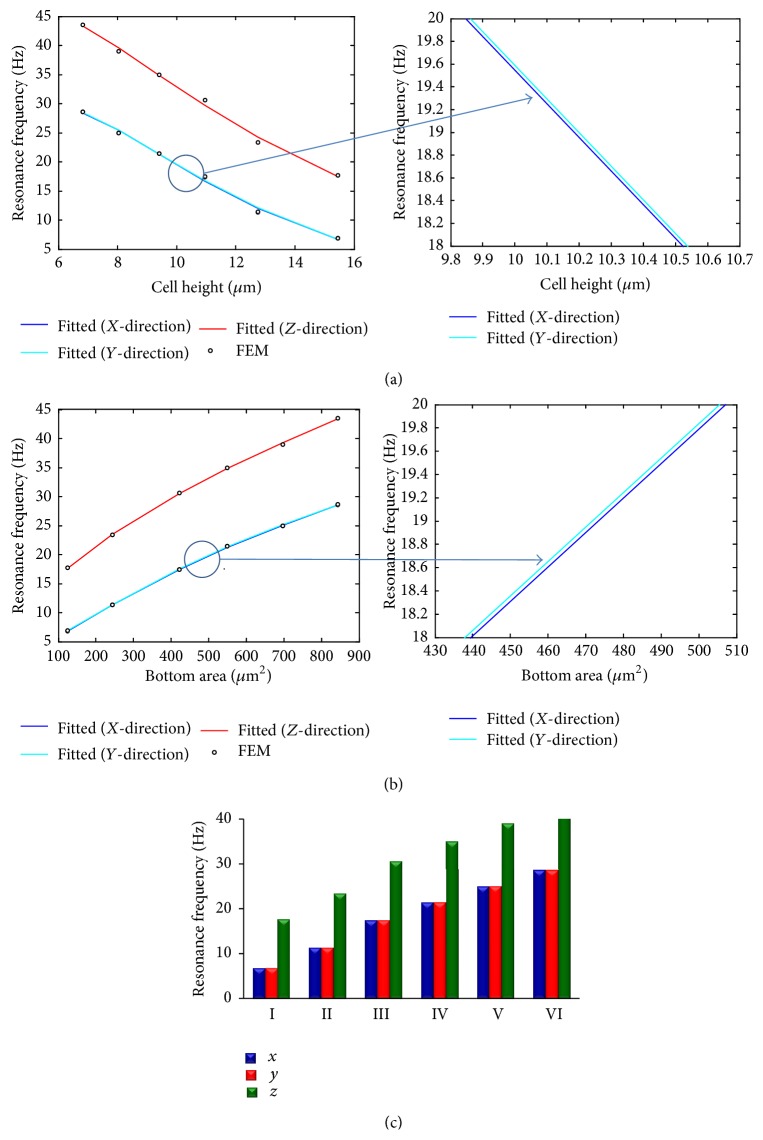
Resonance frequency responses to the osteoblast models in different directions. (a) Resonance frequency versus cell height; (b) resonance frequency versus bottom area of the cell; and (c) resonance frequency versus different models.

The relationships of resonance frequency with cell height and with bottom area were analysed when the volume and density of the cell were assumed as constants. The results are shown in Figures 12(a) and 12(b), respectively. Moreover, the relationship between frequency and different models was also analysed and is shown in Figure 12(c). These results suggest that the resonance frequency depends on cell height and bottom area. If *h* (cell height) or *s* (bottom area) (Figure 2) is an independent variable, the value *f*_*r*_ (resonance frequency) is the function of *h* (cell height) or *s* (bottom area) and can be expressed through a fitted formula in (5) or (6) based on the data of Figures 12(a) and 12(b) as follows:

(5) $$\begin{array}{c} f_{r}=p_{1}\log h+p_{2}{\left(\;\log h\;\right)}^{2}+p_{3}{\left(\;\log h\;\right)}^{3}+p_{4}, \end{array}$$

where *h* is the cell height and the values of *p*_*i*_ are coefficients that are given in Table 3.

(6) $$\begin{array}{c} f_{r}=p_{1}\log s+p_{2}{\left(\;\log s\;\right)}^{2}+p_{3}{\left(\;\log s\;\right)}^{3}+p_{4}, \end{array}$$

where *s* is the bottom area and the values of *p*_*i*_ are coefficients that are given in Table 4.

## 4. Discussion

There have been many previous studies using various models that were developed to reveal bone cell mechanical behaviours. Some numerical computational models of cultured cells have been developed to examine the responses of cultured cells to various mechanical stimulations. For example, to study the universal dynamic behaviours of osteoblasts, a three-dimensional (3D) soft matter cell model was developed using the multiscale moving contact line theory [31, 34]. On the other hand, the finite element (FE) method has been more widely used to analyse the biomechanical behaviours of osteoblasts or other cell types, using the biorealistic or idealized cell models. Based on confocal microscopy data, a 3D cell-specific FE model was created to simulate the cellular mechanics tests such as large deformation [35]. For the idealized FE models, both the continuum model [17, 23, 36] and the tensegrity model [16, 24, 29, 37] have been developed. Although the above prior studies have made significant advancements in revealing bone cell mechanical behaviours, it still remains a great challenge to understand the mechanisms of biomechanical behaviours of osteoblasts in response to vibration signals and their characteristics of deformations under various mechanical stimuli.

In this present study, to investigate the vibrational responses of the different shapes of an osteoblast subjected to vibration of base excitation, six idealized FE models of an osteoblast were created. Firstly, cell geometry was created and FE models were developed accordingly. For these models, the initial volumes of the cell were basically the same and the densities were constant in the simulation. Secondly, natural frequency (resonance frequency) of the different models was extracted by using FE analysis. Then, harmonic vibration of the osteoblast models was analysed with three different base excitation acceleration values, namely, 0.5 g, 1 g, and 2 g. The response results were obtained for the harmonic vibration including displacement, von Mises stress, and strain of the centre of the nucleus under different acceleration values. Finally, the effects of cell height and bottom area on resonance frequency were analysed, and the fitted curves of resonance frequency versus cell height and resonance frequency versus bottom area were obtained.

Based on the previous studies, the vibration frequency is crucial for bone cells to complete the bone resorption and bone formation [1, 11]. Using the FE cell models developed, the natural frequency of osteoblasts was predicted in the current study, with the first ten resonant frequencies of the cells being in the range ~6.85–48.69 Hz. Previously, the vibration frequency was selected as 5–100 Hz for* in vitro* bone cell studies [5, 9]. Similar natural frequency values were computed by other FE studies; for example, the first ten natural frequency values of bone cells were predicted at ~9.95–211.05 Hz for the first ten modes [18] and at ~18.11–21.05 Hz for the first five modes [19]. In comparison with the experimental data and FE studies, the natural frequency of our FE models was within the range in the literatures; the difference may be caused by some factors in FE modelling, for example, the density, material property, and spreading shape. Thus, the FE models developed in the current study can be validated by the data of the literatures.

In this study, the osteoblast models were assumed as one-degree-of-freedom vibrational system. The resonance phenomenon can occur at some natural frequency and can be observed from vibrational responses like displacement, von Mises stress, and strain. The cell biomechanical responses (e.g., displacement, von Mises stress, and strain of the centre of the nucleus models of osteoblasts) were obtained when the models were subjected to three different acceleration values of base excitation vibration (0.5 g, 1 g, and 2 g). The current study observed that the resonance frequency does not change with the acceleration, which suggests that the natural frequency of the bone cell is determined by the intrinsic factors and is not affected by the external factors. The current study has also examined the vibration responses of different FE models of the bone cell under 0.5 g acceleration in *X*-, *Y*-, and *Z*-directions. It was found that the peak response frequency is the same with the first mode frequency for the acceleration in *X*-direction, and the peak response frequency is around at the second mode frequency for the acceleration in *Y*-direction. While the peak response frequency varies with the models for the acceleration in *Z*-direction, the peak frequency occurs round at the fourth mode frequency for Model I to Model IV, sixth mode frequency for Model V, and eighth mode frequency for Model VI. A similar resonance phenomenon was also found by one previous study [19]; it found that the resonance of the continuum model of bone cell occurred at mode 1, mode 2, and mode 3 for *X*-direction, *Y*-direction, and *Z*-direction, respectively. Furthermore, it can be seen from the curves of Figures 6, 9, and 11 that the response peak values at that frequency are remarkably larger than those at the rest. Thus, this phenomenon is typical resonance.

The current study has investigated the displacement, von Mises stress, and strain responses of bone cell models under different acceleration values and in different directions. The results showed that the values of displacement, von Mises stress, and strain increased with the acceleration. The values in *Y*-direction are slightly larger than those in *X*-direction, and the values in *Z*-direction are smaller than those in *X*- and *Y*-directions. Figure 7 presents the displacement response of the different models in *X*-, *Y*-, and *Z*-directions under different acceleration values. Figure 7 further indicates the effect of the acceleration on the displacement response of bone cell for the external vibration.

Our FE models also indicate that von Mises stress is concentrated in the nucleus and strain is basically concentrated around the nucleus for the harmonic response under 0.5 g acceleration in *Z*-direction. By comparing Model I and Model VI at the peak frequency, the maximum von Mises stress value changed from 590.4 Pa (Model I) to 129.5 Pa (Model VI), and the maximum strain value changed from 327300 microstrains (Model I) to 67010 microstrains (Model VI). Therefore, the maximum von Mises stress and the maximum strain values reduce by 78.07% and 78.92%, respectively. They reduce with the decrease of cell height or with the increase of bottom area, and thus the cell shape has a great influence on the harmonic response.

Based on the simulation results analysis, there is a relationship between resonance frequency and cell height or between resonance frequency and bottom area. The resonance frequency can be expressed as the function of cell height (*h*) or bottom area (*s*) (see (5)). The resonance frequency is a monotonic decreasing function of cell height (*h*) and is a monotonic increasing function of bottom area (*s*). In other words, the cell volume and material properties do not vary and* h *or* s* is the independent variable, and the resonance frequency will decrease with the increase of* h* or increase with the increase of* s*. The resonance frequencies in *Z*-direction are much higher than those in *X*- and *Y*-direction, and the resonance frequencies in *X*-direction and *Y*-direction are very close. That is because the bottom areas are assumed as a circle, and the differences of resonance frequencies between *X*- and *Y*-direction would be larger if the bottom areas are not circle but an ellipse.

It must be noted that, in this study, the geometry of the bone cell was assumed to be comprised of three components, that is, membrane, cytoplasm, and nucleus. In the previous studies, the tensegrity structure was used to simulate the fibres of cells like microtubules and microfilaments [29, 37]. In those studies, the nucleus was in the centre of the cell, and the nucleus and the cell membrane were connected by the tensegrity structure (microtubules and microfilaments). However, the effect of microtubules and microfilaments on the harmonic response was not large [19]. For the present FE models, membrane, cytoplasm, and nucleus were assumed to be the linear elastic homogeneous materials. Although the cell has viscoelastic characteristics, which can lead to the damping effect, the viscoelastic material property was ignored in all simulations in this study as the effects of damping on the osteoblast were found to be small [19]. Furthermore, it is well accepted that the stiffness ratio of cytoplasm and nucleus of the cell is 1 : 4 and the elastic modulus of cell membrane is 1 kPa. Further studies using the nonlinear material property and biorealistic geometries are being considered in further investigations.

## 5. Conclusion

In the current study, different osteoblast FE models were developed and were used to extract the natural frequencies and to analyse the harmonic responses under different acceleration values (0.5 g, 1 g, and 2 g). It was found that the natural frequencies do not change with the variation of acceleration of base excitation. The response values of displacement, von Mises stress, and strain increase with the increase of acceleration, and the response values in *Z*-direction are much higher than those in the other directions (*X*- and *Y*-direction). Moreover, the model simulation predicted that the von Mises stress is concentrated in the nucleus and strain is basically concentrated around the nucleus. This study also found that the resonance frequencies can be a monotonic function of cell height or bottom area when the cell volume and material properties are assumed as constants. Therefore, the cell shape has a great influence on the vibrational characteristics of the osteoblast. Therefore, the FE simulations of osteoblast harmonic vibration presented in this study have provided adequate description of vibrational responses of osteoblasts* in vitro*. These findings could be helpful for guiding the* in vitro* cell culture biomechanical research and will help in understanding the deformation of osteoblasts under various mechanical stimuli.

## Figures and Tables

**Table 1:** Geometry property and element data for osteoblast finite element models.

Models	I	II	III	IV	V	VI
Cell height (*μ*m)	15.45	12.75	10.96	9.41	8.03	6.82
Surface (*μ*m^2^)	898.98	835.24	799.52	825.35	891.97	985.89
Bottom area (*μ*m^2^)	125.09	243.84	421.28	549.88	696.53	842.90
Volume (*μ*m^3^)	2988.40	2998.36	3000.60	3000.49	3010.90	3019.02
Nucleus volume (*μ*m^3^)	104.72	104.72	104.72	104.72	104.72	104.72
Number of elements						
Nucleus	20472	20092	20668	25248	23424	17388
Cytoplasm	148020	127884	115060	130880	111808	88592
Membrane	7480	7288	8056	9856	10048	8932

**Table 2:** Material properties and density for the osteoblast finite element models.

Components	Young's modulus	Poisson's ratio	Density
Membrane	1 kPa	0.3	600 kg/m^3^
Cytoplasm	1.5 kPa	0.37	1500 kg/m^3^
Nucleus	6 kPa	0.37	1800 kg/m^3^

**Table 3:** Values of *p*_*i*_.

Independent variable	Coefficient of *p*_*i*_	Direction		
		*X*	*Y*	*Z*
*h* (cell height, *μ*m)	*p*_1_	852.63	856.51	607.60
	*p*_2_	−874.73	−878.29	−628.97
	*p*_3_	275.97	277.04	190.94
	*p*_4_	−234.36	−235.70	−136.72

**Table 4:** Values of *p*_*i*_.

Independent variable	Coefficient of *p*_*i*_	Direction		
		*X*	*Y*	*Z*
*s* (bottom area, *μ*m^2^)	*p*_1_	−50.63	−55.41	61.23
	*p*_2_	10.61	12.57	−34.011
	*p*_3_	1.23	0.96	7.36
	*p*_4_	55.01	58.90	−29.06
